# Spontaneous Fistula and Abdominal Wall Endometriosis Due to Occult Existence of Unicornuate Right Uterus with Rudimentary Non-Communicating Functioning Left Horn

**DOI:** 10.3390/diagnostics14050532

**Published:** 2024-03-02

**Authors:** Gheorghe Cruciat, Adelina Staicu, Andreea Florian, Georgiana Nemeti, Diana Sachelaru, David Andras, Daniel Muresan

**Affiliations:** 1 Obstetrics and Gynecology I, Mother and Child Department, “Iuliu Hatieganu” University of Medicine and Pharmacy, 400012 Cluj-Napoca, Romania; gheorghe.cruciat@elearn.umfcluj.ro (G.C.); florianandreea@ymail.com (A.F.); georgiana.nemeti@elearn.umfcluj.ro (G.N.); dianasachelaru@yahoo.com (D.S.); daniel.muresan@umfcluj.ro (D.M.); 2 Department of Surgery, “Iuliu Haţieganu” University of Medicine and Pharmacy, 400012 Cluj-Napoca, Romania; andrasdavid88@elearn.umfcluj.ro

**Keywords:** abdominal wall endometriosis, Müllerian duct anomaly, rudimentary non-communicating left horn, spontaneous fistula

## Abstract

Accurate diagnosis of Müllerian duct anomalies (MDA) remains a clinical challenge even by direct surgical inspection. Although obstetrical complications are more frequent in women with MDA, some subtypes allow normal reproduction, further delaying the diagnosis. Unicornuate uterus with a rudimentary non-communicating functioning horn is a rare form of MDA, susceptible to many gynecologic and obstetric complications such as miscarriages, premature birth, hematosalpinx, endometriosis, and chronic pelvic pain. We present an entire case pictorial assay including preoperative imaging as well as the surgical correction of the uterine anomaly and the associated complication of an occult unicornuate right uterus with rudimentary non-communicating functioning left horn (Class U4aC0V0/ European Society of Human Reproduction and Embryology/European Society of Gastrointestinal Endoscopy Classification) and its natural evolution following a previous incomplete surgical treatment. The patient had an emergency left adnexectomy for hematosalpinx and ovarian endometrioma at her local county hospital. After five years, the patient presented with severe dysmenorrhea and abdominal endometriosis due to blocked retrograde menstruation from a rudimentary, non-communicating functioning horn. Surgical treatment with the resection of the rudimentary uterine horn, together with the abdominal wall endometriosis lesions, was carried out with good outcomes.

Non-communicating rudimentary horns with a functional endometrium are exceptional Müllerian anomalies in 20% to 25% of women with a unicornuate uterus [1,2]. The exact prevalence of Müller duct anomalies (MDAs) remains unknown because of underdiagnosing and under-reporting. Several patients are asymptomatic and remain undetected despite routine gynecologic screening and even following obstetric management for childbirth and delivery. Common complications of MDAs include hematosalpinx, endometriosis, chronic pelvic pain, and adhesions, all secondary to retrograde menstruation [3]. From the obstetric perspective, most patients have significant obstetric complications, rendering term deliveries exceptional [4]. In asymptomatic women with occult MDAs, the diagnosis is a challenging task during a routine gynecological examination [5]. The detection of MDAs is also a surgical challenge, making the preoperative imaging workup essential—ultrasound, hysteroscopy, and MRI [6,7].

We present a sporadic case of occult unicornuate right uterus with rudimentary non-communicating functioning left horn and its natural evolution following an initially incomplete surgical treatment.

A 30-year-old Caucasian female patient, with a previous term vaginal delivery seven years prior to the current presentation, was referred to our hospital for severe secondary dysmenorrhea and a painful, non-inflammatory Pfannenstiel scar mass with progressively increasing size with passing menses. The patient had undergone classic laparotomy and adnexectomy five years before for an emergency presentation interpreted as hematosalpinx and left ovarian endometrioma at a local county hospital.

Upon clinical examination, we identified a firm abdominal wall mass of approximately 8 cm in length and 5 cm in width in the middle line of the Pfannenstiel scar, with a violaceous appearance, adherent to deep tissues, painful at palpation, and suggestive of parietal endometrioma (Figure 1A). The pelvic examination was unremarkable, with a typical-looking cervix.

Abdominal and three-dimensional transvaginal ultrasound revealed a right unicornuate uterus with proliferative endometrium and a thick-walled left pelvic mass measuring 7 cm high and 5.5 cm wide, suggestive of hematometra in a non-communicating left horn (Figure 1B). Silhouette ultrasound reconstruction of the uterus describes a fistulous communication between the left uterine horn and the anterior abdominal wall mass, with characteristics pointing to an endometrioma (Figure 1C). The right ovary had a typical ultrasound appearance. A minimal fluid collection was noted in the Douglas pouch. A renal ultrasound confirmed a solitary right kidney.

Pelvic magnetic resonance imaging (MRI) revealed a typical-looking right uterus and right ovary and a pelvic mass located mainly to the left, measuring 47/63 mm, with similar enhancement to the uterus, indicative of a Müllerian duct anomaly (MDA) type U4aC0V0 (Class U4aC0V0/European Society of Human Reproduction and Embryology/European Society of Gastrointestinal Endoscopy Classification) [8] or class II-B of the American Society for Reproductive Medicine Classification Scheme [9], with large hematometra and solitary right kidney (Figure 2). A fistulous tract was described between the left hemi-uterine fundus towards the urinary bladder (without its involvement), passing anteriorly through the rectus abdominis muscles, reaching the subcutaneous tissue and the tegument in the scar area (Figure 3). No other endometriosis lesions were noted, and the patient was classified as #Enzian (m) P0, O0/0, B0/0, C0, FU(0), and FI (abdominal wall).

Following patient counseling and approval, a laparotomy was performed. Intraoperatively, extensive adesiolysis was required without evidence of other endometriotic lesions. All lesions described by MRI were confirmed during surgery. The left non-communicating horn was resected together with the abdominal wall endometriosis lesions. The fascial defect was closed with a 10/10 cm low-weight polypropylene mesh (Figure 4B). The abdominal drainage was suppressed after two days postoperatively. The intraoperative and postoperative periods were uneventful. The preoperative and postoperative blood work were within normal ranges. The patient was discharged on the 10th day postoperatively. Histopathological examination of the specimen confirmed adenomyosis in the rudimentary horn and abdominal wall endometriosis (Figure 4C). During the six-month follow-up period, there were no complications, and the patient had no complaints of dysmenorrhea or pelvic pain.

In the case of our patient, the unrecognized uterine anomaly during the emergency surgery remained unresolved, favoring hematometra by preventing the evacuation of menstruating blood from the rudimentary, non-communicating functioning horn and later fistula formation in the weak spot of the uterine horn (potentially the tubal ostium).

After incomplete resection, the literature mentions worsening dysmenorrhea as a result of blocking retrograde menstruation [10]. Also, life-threatening complications like massive hemoperitoneum and ruptured horn due to pregnancy in the rudimentary horn may occur [11], reinforcing the idea of routine excision of the rudimentary horn as a prophylaxis to avoid unwanted medical consequences with a negative impact on the patient’s quality of life [12].

To our knowledge, this is the first spontaneous fistula and abdominal endometriosis depicted after an incomplete surgery of a unicornuate uterus with a rudimentary non-communicating functioning horn.

Our case emphasizes the importance of considering the possible diagnosis of Müllerian duct anomalies in patients with a history of secondary dysmenorrhea and chronic pelvic pain despite an obstetric background. Time should be taken to perform imaging investigations regardless of the potential initial case presentation as an emergency to refine diagnosis and ensure correct management. These abnormalities can lead to significant obstetrical and gynecological complications, such as miscarriages, fetal growth restriction, preterm delivery, endometriosis, or rupture of the rudimentary horn. Accurate diagnosis would avoid improper and unwanted medical consequences and ensure a good quality of life for such patients. The recommended treatment is surgical resection of the rudimentary non-communicating horn.

## Figures and Tables

**Figure 1:**
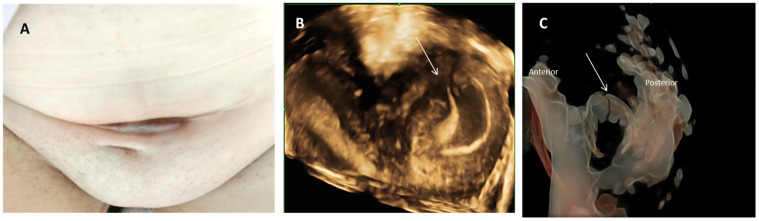
Clinical appearance and ultrasound evaluation: (**A**) Clinical exam at admission revealed in the middle portion of the Pfannenstiel scar a complex mass with a violaceous color. (**B**) Three-dimensional transvaginal ultrasound revealed a unicornuate uterus and a thick-walled left pelvic mass suggestive of hematometra in a non-communicating left horn (white arrow). (**C**) Silhouette ultrasound reconstruction of the uterus describing a fistulous communication between the left uterine horn and the anterior abdominal wall mass.

**Figure 2:**
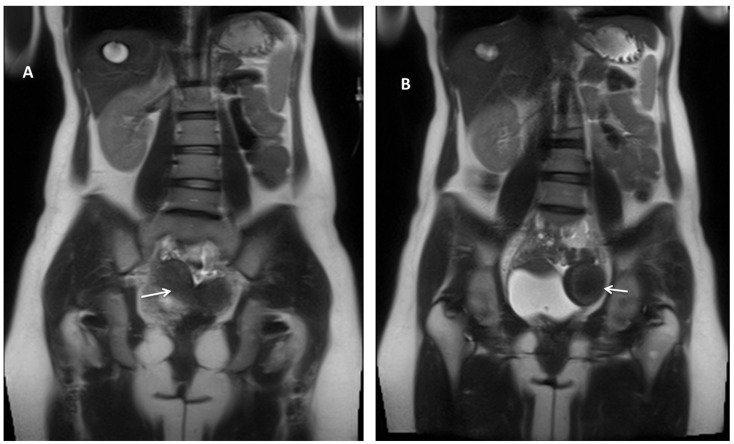
Abdominal and pelvic MRI, coronal T2-weighted images. Pelvic MRI revealed (**A**) a typical-looking right uterus (white arrow) and right ovary and (**B**) a pelvic mass located mainly to the left (white arrow) with similar enhancement to the uterus, indicative of a Müllerian duct anomaly type U4aC0V0 with large hematometra and solitary right kidney and absent left kidney.

**Figure 3:**
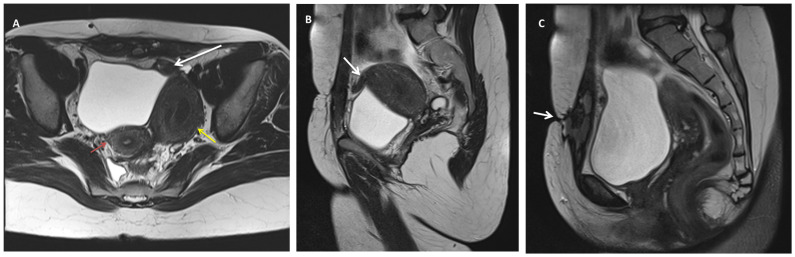
Pelvic MRI T2-weighted images depicting (**A**) axial view of a unicornuate right uterus (red arrow) and a large non-communicating cavitary left horn (U4a) with hematometra (yellow arrow). (**B**) sagittal view (white arrow) The white arrow is pointing to a fistulous trajectory towards anterior and medial directions. (**C**) that drains in the rectus abdominis muscles and subcutaneous tissue, reaching the scar tegument (arrow).

**Figure 4:**
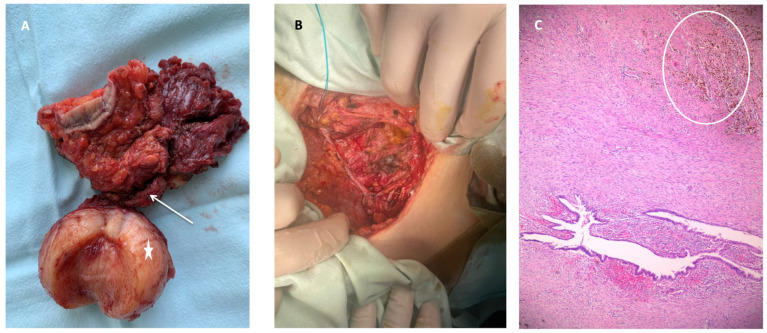
Intraoperative and microscopic confirmation: (**A**) Surgical excision specimen. The arrow points to the fistulous trajectory from the rudimentary uterus (star) (cut medially to demonstrate the endometrial cavity). (**B**) Post-surgical excision fascial defect. (**C**) HE 5X, depicting endometriosis lesions in the muscular tissue (circle).

## Data Availability

No new data were created.
